# Interleukin-1β Mediates Arterial Thrombus Formation via NET-Associated Tissue Factor

**DOI:** 10.3390/jcm8122072

**Published:** 2019-11-26

**Authors:** Luca Liberale, Erik W. Holy, Alexander Akhmedov, Nicole R. Bonetti, Fabian Nietlispach, Christian M. Matter, François Mach, Fabrizio Montecucco, Jürg H. Beer, Francesco Paneni, Frank Ruschitzka, Peter Libby, Thomas F. Lüscher, Giovanni G. Camici

**Affiliations:** 1 Center for Molecular Cardiology, Schlieren Campus, University of Zurich, 8952 Schlieren, Switzerland; luca.liberale@uzh.ch (L.L.); alexander.akhmedov@uzh.ch (A.A.); nicole.bonetti@uzh.ch (N.R.B.); christian.matter@uzh.ch (C.M.M.); francesco.paneni@uzh.ch (F.P.); thomas.luescher@zhh.ch (T.F.L.); 2 First Clinic of Internal Medicine, Department of Internal Medicine, University of Genoa, 16132 Genoa, Italy; fabrizio.montecucco@unige.it; 3 Department of Cardiology, University Hospital Zurich, 8091 Zurich, Switzerland; erik.holy@usz.ch (E.W.H.); fabian.nietlispach@usz.ch (F.N.); frank.ruschitzka@usz.ch (F.R.); 4 Department of Internal Medicine, Cantonal Hospital of Baden, 5404 Baden, Switzerland; 5 Department of Cardiology, Hopital Universitaire de Geneve, 1206 Geneve, Switzerland; francois.mach@unige.ch; 6 IRCCS Ospedale Policlinico San Martino Genoa—Italian Cardiovascular Network, 16132 Genoa, Italy; 7 Department of Research and Education, University Hospital Zurich, 8001 Zurich, Switzerland; 8 Division of Cardiovascular Medicine, Department of Medicine, Brigham and Women’s Hospital, Harvard Medical School, 02115 Boston, MA, USA; plibby@bwh.harvard.edu; 9 Royal Brompton and Harefield Hospitals and Imperial College, London SW3 6NP, UK

**Keywords:** IL-1β, Canakinumab, arterial thrombosis, tissue factor, neutrophil extracellular traps

## Abstract

CANTOS reported reduced secondary atherothrombotic events in patients with residual inflammatory risk treated with the inhibitory anti-IL-1β antibody, Canakinumab. Yet, mechanisms that underlie this benefit remain elusive. Recent work has implicated formation of neutrophil extracellular traps (NETosis) in arterial thrombosis. Hence, the present study explored the potential link between IL-1β, NETs, and tissue factor (TF)—the key trigger of the coagulation cascade—in atherothrombosis. To this end, ST-elevation myocardial infarction (STEMI) patients from the Swiss multicenter trial SPUM-ACS were retrospectively and randomly selected based on their CRP levels. In particular, 33 patients with STEMI and high C-reactive protein (CRP) levels (≥ 10 mg/L) and, 33 with STEMI and low CRP levels (≤ 4 mg/L) were investigated. High CRP patients displayed elevated circulating IL-1β, NETosis, and NET-associated TF plasma levels compared with low CRP ones. Additionally, analysis of patients stratified by circulating IL-1β levels yielded similar results. Moreover, NETosis and NET-associated TF plasma levels correlated positively in the whole population. In addition to the above, translational research experiments provided mechanistic confirmation for the clinical data identifying IL-1β as the initial trigger for the release of the pro-coagulant, NET-associated TF. In conclusion, blunted TF presentation by activated neutrophils undergoing NETosis may provide a mechanistic explanation to reduced secondary atherothrombotic events as observed in canakinumab-treated patients in CANTOS.

## 1. Introduction

The Canakinumab Anti-inflammatory Thrombosis Outcomes Study (CANTOS) [1] affirmed in humans the inflammatory theory of atherosclerosis [2]. In CANTOS, over 10,000 post-myocardial infarction patients with residual inflammatory risk (defined as high-sensitivity C-reactive protein (hs-CRP) concentrations ≥ 2 mg/L) and treated with currently mandated concomitant cardiovascular (CV) therapy randomly received placebo or one of 3 doses of the interleukin (IL)-1β neutralizing antibody canakinumab. Canakinumab treatment reduced the primary composite endpoint (non-fatal myocardial infarction or stroke and cardiovascular death) by 15%, and blunted circulating levels of hsCRP and IL-6 by 35–40% without lowering atherogenic lipids [1]. Yet, the molecular mechanisms underlying this clinical benefit, particularly its thrombotic component, remain incompletely understood.

Occlusive arterial thrombus formation following plaque rupture or erosion causes most myocardial infarctions and many ischemic strokes [3]. Several experimental observations suggest a strong bidirectional link between inflammation, atherosclerosis and arterial thrombosis [4,5,6,7,8]. Indeed, inflammatory conditions favour atherothrombosis as reflected by increased plaque burden and CV event rates in patients with chronic inflammatory diseases [9]. Moreover, thrombosis itself can also induce and enhance inflammation thus setting the stage for a vicious cycle involving different pathways, and more prominently the tissue factor (TF)-thrombin axis [10]. Recent work has implicated neutrophil extracellular traps (NETs) in thrombosis by various mechanisms including presentation of tissue factor procoagulant activity [11,12,13]. Formation of NETs contributes to the host defence properties of neutrophils. Various infectious and non-infectious stimuli induce NETs which consist of extracellular strands of unwound DNA in complex with histones and proteins from neutrophil granules [13,14].

Hence, the present study explored the molecular mechanisms by which canakinumab reduces atherothrombotic events, as observed in CANTOS. To this end, we tested the hypothesis that IL-1β, NETosis, and TF-bearing NETs interrelate in a cohort of patients with ST-elevation myocardial infarction (STEMI) and different degree of systemic inflammation randomly selected from the Swiss SPUM-ACS trial (ClinicalTrials.gov number NCT01000701) [15]. To provide mechanistic explanations for these clinical findings, mouse experiments used a murine canakinumab-surrogate antibody (01BSUR) IL-1β to inhibit this cytokine in lipopolysaccharide (LPS)-treated mice before induction of arterial thrombosis by endothelial-specific photochemical injury.

## 2. Experimental Section

### 2.1. Patient Enrollment and Blood Sampling

The SPUM–ACS study (Special Program University Medicine–Acute Coronary Syndromes) is a prospective cohort study of consecutive acute coronary syndrome (ACS) patients hospitalized in different Swiss university hospitals registered in ClinicalTrials.gov (NCT01000701). Details of the study have been reported previously [15]. Briefly, patients with ACS undergoing coronary angiography were consecutively enrolled in four Swiss medical centres between December 2009 and October 2012. Exclusion criteria were severe physical disability, inability to give consent owing to dementia, and life expectancy of <1 year for non-cardiac reasons. Inclusion criteria were age ≥18 years, ST-segment elevation myocardial infarction (STEMI), non-ST-segment–elevation myocardial infarction, or unstable angina. The local ethics committees approved the study and all patients gave written informed consent in compliance with the Declaration of Helsinki as listed under ClinicalTrials.gov number NCT01000701.

STEMI patients were enrolled at the Andreas-Grüntzig-Catheterisation laboratory of the Department of Cardiology at the University Hospital Zurich, Switzerland, within 72 h after pain onset, and subdivided into two groups. Specifically, plasma samples of 33 STEMI patients with high circulating C-reactive protein (CRP) levels ≥ 10 mg/L and 33 STEMI patients with low CRP levels ≤ 4 mg/L and were analysed. Liver or kidney failure, active cancer, infections, or autoimmune disease were exclusion criteria. Blood was drawn from the arterial sheath into EDTA tubes at the time of diagnostic coronary angiography and centrifuged at 2700 g for 10 min at room temperature to obtain plasma, and frozen and stored in aliquots at −80 °C until serial measurement in the core laboratory (Department of Clinical Chemistry, University Hospital Zurich, Switzerland).

### 2.2. IL-1β Quantification in Human Plasma

Plasma levels of IL-1β were measured by high-sensitivity ELISA following the manufacturer’s instructions (Quantikine^®^ HSLB00D, R&D Systems, Minneapolis, MN). Plasma samples with an absorbance value below the lowest IL-1β concentration included in the standard curve (assessed in duplicates) were assigned an absorbance equal to that of the lowest point of the standard curve.

### 2.3. NETosis Assessment in Human and Murine Plasma

Quantification of circulating NET remnants such as myeloperoxidase (MPO)-DNA complexes by ELISA appears to be the current, most specific and objective assay to monitor NETosis in vivo [12,16,17]. As previously described [12,17], microtiter plates were coated with anti-MPO antibody (5 μg/mL, Cat No. 07-496, Merck, Darmstadt, Germany) overnight at 4 °C. After blocking with 1% BSA, the serum was added in combination with the peroxidase-conjugated anti-DNA monoclonal antibody (part of the commercial cell death detection kit; Roche, Basel, Switzerland) according to the manufacturer’s instructions. After 2 h of incubation and accurate washing with PBS, the peroxidase substrate (ATBS) from the kit was added to each plate. The absorbance at 405 nm wavelength was measured after 40 min incubation in the dark, 490 nm was used as reference wavelength.

### 2.4. Tissue Factor-DNA Complexes Quantification in Human and Murine Plasma

To assess the NET contribution to circulating TF levels, we sought to quantify the fraction of TF associated with circulating DNA. This protocol adapted that used for MPO-DNA complex assessment to detect TF–DNA complexes. Briefly, microtiter plate was coated with either human or mouse TF capture antibody (parts of the commercial DuoSet^®^ ELISA kits from R&D Systems, Cat No. DY2339 and DY3178-05, respectively) according to the manufacturer’s instructions. After overnight incubation and blocking with 1% BSA, serum was added in combination with the peroxidase-conjugated anti-DNA monoclonal antibody (component No.2 of the commercial cell death detection kit from Roche, cat No. 11774425001) following the manufacturer’s instructions. Validation experiments measured MPO-DNA and TF–DNA complexes in human plasma incubated with increasing concentration of LPS to stimulate NETosis (Supplemental Figure S1). Briefly, blood was collected in EDTA tubes (Vacutainer, BD Diagnostics) from a healthy donor and aliquoted in tubes containing increasing concentration of LPS dissolved in PBS (0–25 μg/mL) [18]. After 2 h, plasma was isolated by centrifugation at 2700 g for 15 min and MPO-DNA and TF–DNA complexes assessed as mentioned above (Supplemental Figure S1).

### 2.5. Animals

Experiments used 12-week-old male C57BL/6 wild-type mice (Charles-River Lab, Freiburg im Breisgau, Germany); all rodents were kept in a temperature-controlled animal facility under normal light/dark cycle with free access to food and water. All procedures were approved by the Committee for Animal Testing of the Canton of Zurich, Switzerland (ZH023/17). Animal experiments were performed conform to the Directive 2010/63/EU of the European Parliament and of the Council of 22 September 2010 on the protection of animals used for scientific purposes.

### 2.6. Monoclonal anti IL-1β Antibody

Dr. Hermann Gram (Novartis, Basel, Switzerland) kindly provided the highly specific canakinumab-surrogate anti-mouse IL-1β antibody 01BSUR. Since the human anti IL-1β antibody (i.e., canakinumab) does not neutralize the rodent antigen, experiments used this monoclonal anti-IL-1β antibody instead. Novartis used 01BSUR, a murine IgG2a/k isotype, in all parallel pre-clinical studies performed for the development of canakinumab [19,20].

### 2.7. Treatments and Arterial Thrombosis

As previously described [21], mice received 5 mg/kg lipopolysaccharide (LPS) (E. coli O111: B4, Sigma–Aldrich, St. Louis, MO, USA) by intraperitoneal (i.p.) injection 10 h before the laser-induced arterial thrombosis to trigger inflammation. Five hours after LPS administration and for the remaining 5 h before exposure to the thrombosis protocol, animals randomly received the murine canakinumab-surrogate antibody (01BSUR, 10 µg/g) or vehicle (i.e., NaCl 0.9%) via tail vein injection. The unique dose was chosen to reproduce the canakinumab concentration in sera from patients enrolled in the clinical trial CACZ885A2102 (NCT00487708), as previously described [20,22,23,24,25].

**Figure 1:**
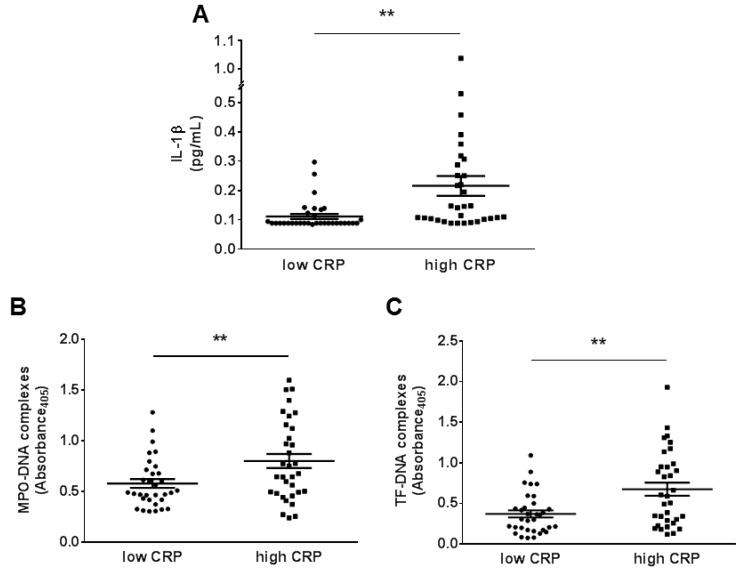

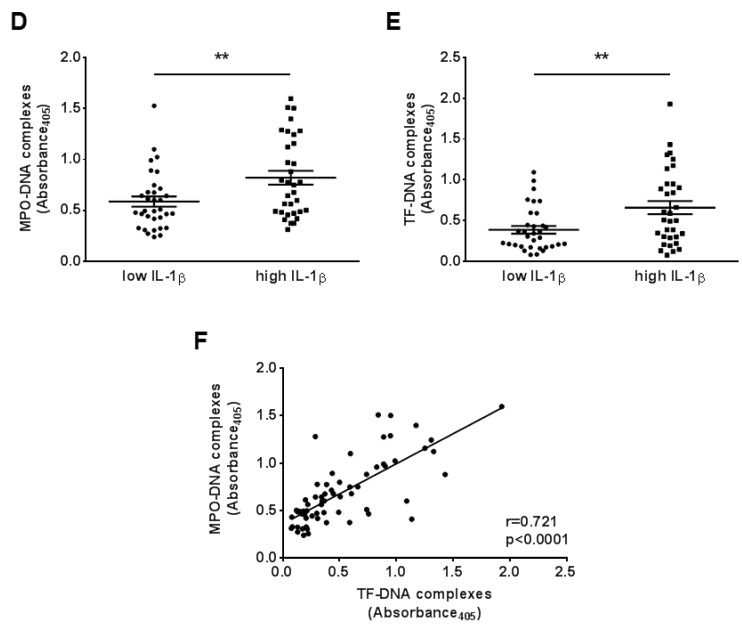
Levels of IL-1β, neutrophil extracellular traps (NETosis), NET-associated tissue factor and their relationships in patients with coronary disease. (**A**) STEMI patients with high systemic inflammation have higher IL-1β plasma levels than those with lower C-reactive protein (CRP) levels (*n* = 33). (**B**) Patients with STEMI and high CRP levels showed increased levels of NETosis assessed by plasma MPO-DNA complexes (*n* = 32–33) (**C**) Plasma tissue factor TF)-DNA complexes rose in patients with STEMI and high circulating CRP compared to less inflamed patients (*n* = 33). (**D–E**) Similarly, in patients categorized according to circulating IL-1β levels (above or below the median value of our cohort 0.1041 pg/mL), those with high levels of this cytokine also showed increased myeloperoxidase (MPO)-DNA and TF–DNA circulating complexes (*n* = 33) (**F**) A positive relationship was detectable among plasma IL-1β and MPO-DNA complexes in the whole cohort (*n* = 66). **p < 0.01. CRP = C reactive protein, IL-1β = interleukin-1β, MPO = myeloperoxidase, STEMI = ST-elevated myocardial infarction, TF = tissue factor.

Ten hours after i.p. LPS injection and five hours after i.v. treatment with the canakinumab-surrogate antibody, mice underwent photochemical injury of the common carotid artery CCA as previously described (Figure 1A) [26,27,28]. Briefly, mice were anaesthetized using pentobarbital (87 mg/kg body weight); after midline neck incision, the right common carotid artery was exposed under an operating microscope. To induce photochemical injury of the endothelium, rose bengal (50 mg/kg body weight) was injected into the tail vein and the common carotid artery was exposed to a laser light beam (1.5 mW, 540 nm, Mellesgriot Inc., Carlsbad, CA, USA) at a distance of 6 cm for 60 min. Carotid blood flow and heart rate were monitored (Doppler flow probe carotid artery Transonic Systems Inc., 0.5 VB) until occlusion (flow ≤0.1 mL for 1 min) or for a maximum of 120 min, in case arterial thrombosis was not detected.

### 2.8. Artery and Plasma Sampling for Tissue Factor Assessment

Aortas were collected after euthanasia and snap frozen in liquid nitrogen. They were subsequently homogenized in the lysis buffer (Tris 50 mM, NaCl 150 mM, EDTA 1 mM, NaF 1 mM, DTT 1 mM, aprotinin 10 mg/mL, leupeptin 10 mg/mL, Na3VO4 0.1 mM, phenylmethylsulfonyl fluoride (PMSF) 1 mM, and NP-40 0.5%); next, total protein concentration was determined by Bradford protein assay according to the manufacturer’s recommendations (VWR Life Science AMRESCO, Solon, OH, USA). Blood was collected via intracardiac puncture and immediately mixed with EDTA. The EDTA-blood solution was then centrifuged for 15 min at 3000 g. Plasma was collected and snap-frozen in liquid nitrogen. TF protein measurement used a colorimetric enzyme-linked immunosorbent assay (ELISA) specific for the mouse protein (DY3178-05; R&D systems, Minneapolis, MN, USA) following the manufacturer’s instructions. For aortas, TF concentration measured by ELISA was normalized to the total protein content of the sample. TF content was expressed as pg/mg of total protein.

### 2.9. Statistical Analysis

Data are expressed as mean ± SEM. All statistical analyses used GraphPad Prism 6 software (GraphPad Software, Inc, La Jolla, CA, USA). Data were analysed by one-way analysis of variance (ANOVA) with Bonferroni *post hoc* test for multiple comparisons or unpaired two-tailed Student’s *t*-test as appropriate. Fisher’s exact test was used for comparison of categorical data between study subjects, and Pearson’s correlation analysis was used to test the correlation between two quantitative variables. A probability *p* value below 0.05 was considered as statistically significant.

## 3. Results

### 3.1. IL-1β levels, NETosis and TF–DNA Complexes Increase in STEMI Patients with High Systemic Inflammation

To explore the relationship between IL-1β, NETosis, and TF-bearing NETs we have retrospectively and randomly selected patients with STEMI and different degree of systemic inflammation (CRP ≤ 4 mg/L or ≥ 10 mg/L) from the Swiss SPUM-ACS trial. Then, IL-1β levels as well as circulating MPO-DNA and TF–DNA complexes were measured by ELISA and compared between the high CRP and low CRP groups.

66 STEMI patients (33 for each degree of inflammation group) with a median age of 66 years were enrolled. Table 1 reports a complete assessments of demographic and clinic characteristics of the whole cohort. As expected, patients were at high cardiovascular risk with 65.2% of them being active smokers, 50.0% suffering from hypertension and another 50% showing dyslipidaemia. Ongoing medical therapy at the time of angiography (i.e., anti-platelets, diuretic, anti-hypertensive or lipid-lowering drugs) is detailed in Table 1. Of importance, the two groups did not differ in blood pressure, BMI, smoking habits, comorbidities (i.e., dyslipidaemia and diabetes mellitus) or medications.

Circulating IL-1β levels increased significantly in STEMI patients with high CRP concentrations as compared to those with lower CRP (0.215 ± 0.034 vs. 0.111 ± 0.009 pg/mL; *p* < 0.01; Figure 1A). Circulating plasma MPO-DNA complexes were also higher in the high CRP group compared to patients with low CRP (0.80 ± 0.07 vs. 0.58 ± 0.04; *p* < 0.01; Figure 1B). Furthermore, patients with STEMI and elevated CRP had statistical significantly higher levels of circulating TF–DNA complexes than those with low CRP levels as compared the less inflamed subjects (0.67 ± 0.08 vs. 0.37 ± 0.04; *p* < 0.01; Figure 1C). Categorization of patients according to circulating IL-1β levels (above or below its median value in the cohort: 0.1041 pg/mL) yielded similar results to those observed with lower and higher CRP cut-off points as defined above (0.821 ± 0.07 vs. 0.586 ± 0.05 for MPO-DNA and 0.66 ± 0.08 vs. 0.39 ± 0.05 for TF–DNA; *p* < 0.01 for both; Figure 1D–E). Moreover, a strong positive correlation was observed in the entire STEMI cohort between the NETosis marker MPO-DNA and NET-associated TF in plasma (r = 0.721; *p* < 0.001; Figure 1F).

### 3.2. IL-1β Neutralization Delays Arterial Thrombotic Occlusion In Vivo in LPS-Treated Mice

**Figure 2:**
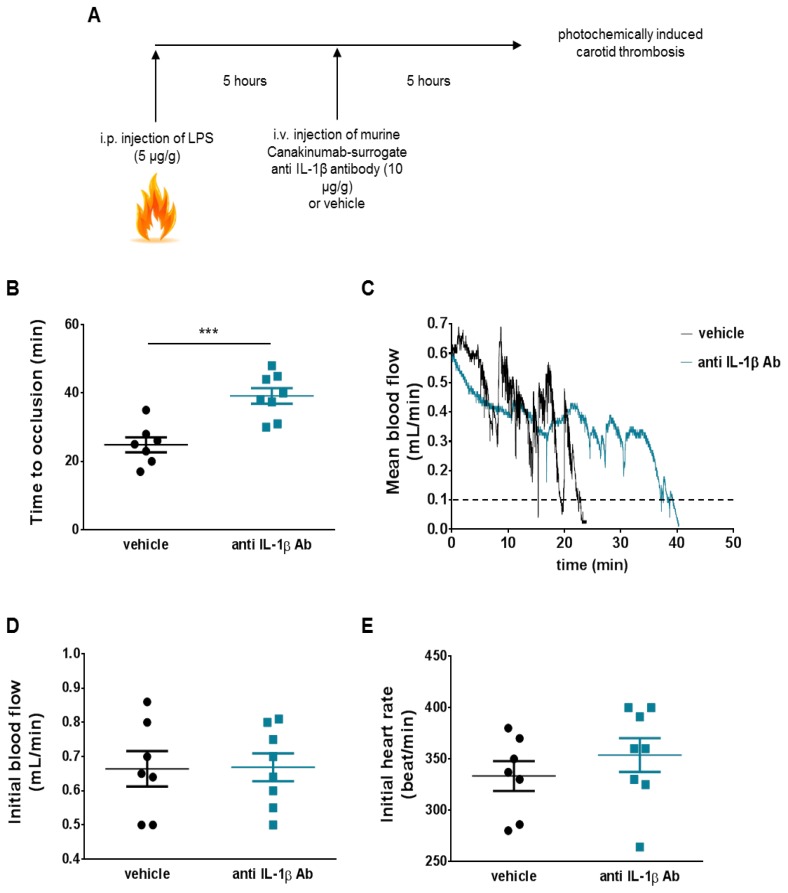
Anti-IL-1β antibody retards arterial thrombosis. (**A**) In an attempt to simulate the design of Canakinumab Anti-inflammatory Thrombosis Outcomes Study (CANTOS), which enrolled patients with residual inflammatory risk, animals received lipopolysaccharide (LPS) (5 μg/g, i.p) 10 h before undergoing photochemically induced carotid thrombosis. The anti-mouse IL-1β monoclonal antibody was administered to the animal intravenously via tail vein injection 5 h before thrombosis at a single dose of 10 µg/g, vehicle (i.e., NaCl 0.9%) was used as negative control. (**B**) Animals treated with monoclonal antibody against IL-1β (anti IL-1β Ab) showed increased time to occlusion as compared to vehicle-treated ones (*n* = 7–8). (**C**) Representative trace of mean blood flow until occlusion (mean flow ≤ 0.1 mL for 1 min) in the two study groups. (**D**,**E**) Baseline blood flow and heart rate did not differ among treated and untreated animals (*n* = 7–8). ***p < 0.001. IL-1β = interleukin-1β, LPS = lipopolysaccharide.

Murine thrombosis experiments sought to mimic an underlying inflammatory condition by administering LPS, as done previously [21]. Next, animals received the canakinumab-surrogate antibody (01BSUR, 10 µg/g) and underwent the thrombosis protocol (Figure 2A). This pre-treatment resembles the situation of recurrent events in CANTOS participants, who were stable at time of enrollment (≥ 30 d post qualifying event.). Compared to controls, inflamed mice treated with anti-IL-1β antibody showed a 50% increase in time to occlusion (24.9 ± 2.2 vs. 39.2 ± 2.3 min, *p* < 0.001, Figure 2B,C). The two study groups had comparable initial mean flow and heart rates (0.66 ± 0.05 vs. 0.67 ± 0.04 mL/min and 333 ± 15 vs. 354 ± 16 beats/min, respectively; Figure 2D,E)

### 3.3. Treatment with Canakinumab-Surrogate Anti-Mouse IL-1β Antibody Reduces Plasma Levels of Tissue Factor

**Figure 3:**
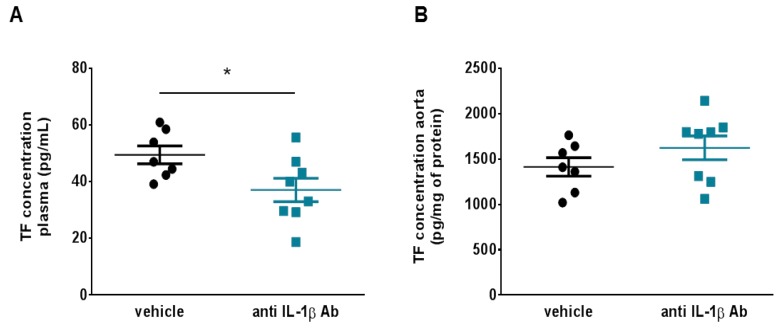
IL-1β blockade reduces different tissue factor pools in mice with arterial thrombosis. (**A**) Treatment with the canakinumab-surrogate antibody (anti IL-1β Ab) reduced plasma TF levels as assessed by ELISA (*n* = 7–8). (**B**) Treated and untreated animals showed no difference in terms of TF concentration in aorta lysates (*n* = 7). * *p* < 0.05. ELISA = enzyme-linked immunosorbent assay, IL-1β = interleukin-1β, TF = tissue factor.

To investigate the mechanisms that underlie the decreased thrombotic potential observed in canakinumab-surrogate treated mice, plasma and aortic lysates were analysed for TF levels, the key trigger of the extrinsic coagulation cascade. TF plasma levels decreased in mice treated with the anti-IL-1β antibody (37.07 ± 4.14 vs. 49.49 ± 3.17 pg/mL; Figure 3A) as compared to controls. On the other hand, TF levels in aortic extracts from the two treatment arms did not differ (1413 ± 101.8 vs. 1623 ± 130.8 pg/mg of protein; Figure 3B).

### 3.4. Anti-IL-1β Treatment Reduces NETosis and NET-Associated Tissue Factor Levels

**Figure 4:**
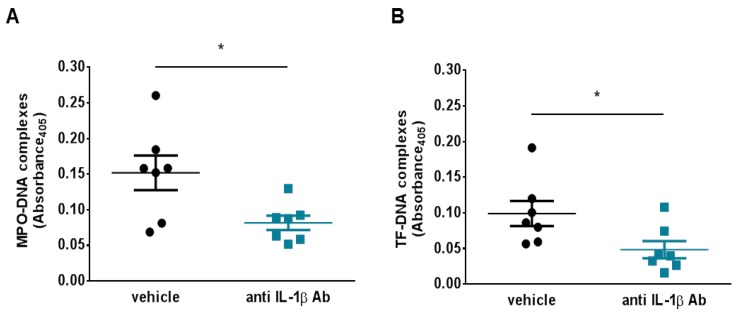
IL-1β blockade limits NETosis and NET-associate tissue factor levels. (**A**) Animal treated with the murine anti IL-1β antibody showed a significant reduction in the level of NETosis plasma marker MPO-DNA complexes (*n* = 7) (**B**) Levels of NET-associated TF fell after thrombosis in animals treated with the anti-IL-1β antibody as assessed by plasma TF–DNA complexes (*n* = 7). * *p* < 0.05. IL-1β = interleukin-1β, MPO = myeloperoxidase, NET = neutrophil extracellular trap, TF = tissue factor.

Since arterial TF levels did not differ between treated and untreated animals, the current study focused on circulating protein as a possible mediator of the retarded thrombosis post IL-1β inhibition. Several cell types contribute to the circulating TF pool during arterial thrombosis. Indeed, NETs can promote thrombus formation by presenting active TF in the setting of myocardial infarction.^12^ Animals treated with anti-IL-1β antibody have reduced levels of circulating MPO-DNA complexes (a marker of in vivo NETosis) after thrombus formation, as compared to vehicle-treated animals (0.082 ± 0.010 vs. 0.152 ± 0.024; *p* < 0.05; Figure 4A). To determine whether the reduced circulating TF levels observed in the treatment group related directly to the reduced NETosis, TF–DNA complexes were assessed in plasma from the two treatment arms. IL-1β inhibition significantly reduced levels of TF–DNA complexes after thrombus formation, as compared to control treatment (0.048 ± 0.012 vs. 0.099 ± 0.018; *p* < 0.05; Figure 4B). Blood cell counts did not differ in the two study subgroups (Table 2).

## 4. Discussion

Ischaemic heart disease and stroke caused by atherothrombotic complications lead global causes of morbidity and mortality triggered by inflammatory bursts [29,30,31]. Recently, CANTOS established a causal role of inflammation in humans with previous ACS and residual inflammatory risk by showing reduced recurrent cardiovascular events after treatment with canakinumab (an anti-IL-1β antibody) [1]. The present study probed the potential mechanisms by which canakinumab may reduce thrombotic events.

This study tested the hypothesis that IL-1β influences NET formation and NETs’ association with tissue factor (TF) the key trigger of the coagulation cascade in atherothrombosis. To assess the role of inflammation, ACS patients with different degree of systemic inflammation—so as to simulate the conditions observed in patients enrolled in CANTOS—were selected. In particular, 33 patients with STEMI and high C-reactive protein (CRP) levels (≥ 10 mg/L) and, 33 with STEMI and low CRP levels (≤ 4 mg/L), were investigated.

Patients were retrospectively and randomly selected from the Swiss multi-center trial SPUM-ACS (*n* = 1639, NCT01000701). The SPUM–ACS study (Special Program University Medicine–Acute Coronary Syndromes) is a prospective cohort study of consecutive acute coronary syndrome (ACS) patients hospitalized in different Swiss university hospitals. Details of the study have been reported previously [15]. Previous investigations have implicated NETs in coronary artery disease [14,32], and the present report correlates systemic inflammation and IL-1β with MPO-DNA complexes, currently considered an established assay for NETosis in vivo [16]. We further assessed circulating NETs-associated TF by measuring plasma TF–DNA complexes and documented their significant elevation in STEMI patients with high CRP plasma levels compared to those with lower CRP. Additionally, categorization of patients according to circulating IL-1β levels showed higher NETosis rate and levels of TF–DNA complexes in patients with higher levels of this cytokine. Moreover, the levels of NET-associated TF correlated with MPO-DNA circulating levels, a novel finding that indicates their strong interdependency. Stakos et al. recently reported NET-associated TF isolated from the culprit coronary artery of ACS patients [12]. Furthermore, these authors found that TF required the intact NET scaffold to be biologically active [12]. The relationship reported here between levels of systemic inflammation, IL-1β, NETosis and NET-associated TF production indicates a common regulating pathway and supports the hypothesis of TF arising from neutrophil undergoing NETosis, as a possible mediator of the benefits of canakinumab treatment.

Experimental data have long suggested a role for IL-1β in atherothrombosis [33,34]; indeed, IL-1β causes vascular smooth muscle cell dysfunction, activates endothelial cells and induces pro-coagulant and adhesion protein expression [35,36]. Furthermore, experimental and human atherosclerotic plaques contain IL-1β and its loss of function limits experimental atherosclerosis [37,38]. Similarly, CANTOS demonstrated that IL-1β blockade reduces recurrent myocardial infarction in human after ACS [1]. The current study sought to experimentally investigate the mechanisms of this effect and showed blunted thrombosis in inflamed mice treated with canakinumab surrogate to resemble the IL-1β-mediated inflammatory risk of patients enrolled in CANTOS.

Arterial thrombosis mediated by activation of platelets and TF causes most ACS [39]. Patients with CV risk factors as well as those with ACS have increased levels of TF [40,41,42]. Various cell types produce TF including endothelial cells, monocytes/macrophages and smooth muscle cells in response to different pro-inflammatory cytokines, among which IL-1β is one of the best-characterized [43,44,45]. In light of the above, we hypothesized that treatment with IL-1β antibody could reduce thrombus formation by targeting TF in vascular and/or circulating cells. IL-1β neutralization in mice reduced TF levels in plasma but not in arterial tissue, suggesting that in our experimental setting IL-1β inhibition primarily targets circulating TF.

As recently reported, activated neutrophils can contribute to blood-borne TF and thrombus formation by binding the injured endothelium [10,46]. Although neutrophils contain TF, either endogenous or acquired via microparticle internalization, questions remained on how these cells release it [10]. In 2012, Kambas et al [47]. reported that neutrophils can release large amounts of TF in the form of NETs. They further showed that NET-associated TF indeed derives from neutrophils, and that the thrombin eventually formed can activate platelets. The same authors reported a requirement for neutrophil priming with pro-inflammatory cytokines (i.e., IL-1β and TNF-α) for TF mRNA translation [47]. In line with the above, the current study found blunted NETosis (MPO-DNA complexes) in IL-1β-blocked animals after thrombus formation, thus underscoring the important role of IL-1β in priming neutrophils for NETosis during inflammatory conditions [48]. Next, we assessed whether blunted NETosis could account for the reduction in TF levels as seen in anti-IL-1β-treated animals by measuring TF–DNA complex in the plasma. Affirming our hypothesis, animals pretreated with IL-1β antibody showed significantly reduced NET-associated TF levels compared to controls. A possible role for IL-1β in NETosis induction has long been hypothesized [49] and recently confirmed in a publication by Meher et al [50]. Here, the authors report that IL-1β localizes within NETs in mice with experimental abdominal aortic aneurysms and that IL-1β blockade inhibits NETosis in isolated neutrophils [50]. They proposed a mechanism by which IL-1β–induced ceramide synthesis triggers the release of NETs by inducing nuclear Cer 16:0 synthesis, which permeabilizes the nuclear membrane and triggers NETosis. Accordingly, attenuation of NETosis can result from either inhibition of IL-1β signaling (IL-1 receptor antagonist treatment) or inhibition of ceramide synthases (fumonisin B1 treatment) [50]. Furthermore, we recently investigated IL-1 isoform association with NETs [51]. Curiously, we found that NET-associated cathepsin G degrades IL-1β to inactive fragments, but actually cleaves IL-1α to a more active form. The IL-1α isoform rather than IL-1β isoform accounts for activation of tissue factor expression and activity on human endothelial cells by NETs [51]. Thus, canakinumab might reduce thrombosis rate by blunting IL-1β-mediated NETosis synergistically with the reduction of other known IL-1β pro-thrombotic features such as its agonist role on platelets [52].

The current study has some limitations which merit consideration. First, patients enrolled in this clinical cohort had less use of CV medications at baseline than in CANTOS, as would be expected in a group that included individuals with a first MI, in contrast to the secondary prevention population in CANTOS. Animal experiments permit in-depth investigation of molecular mechanisms but do not necessarily faithfully replicate the clinical setting. CANTOS enrolled patients with a previous ACS with comorbidities and treated with many medications, conditions that do not apply to the rodents used in this study. Also, while CANTOS reported a reduction of secondary CV events, we here show a reduction of the thrombotic potential in the non-atherosclerotic mouse carotid arteries. Lastly, to mirror the pro-inflammatory state of the CANTOS cohort, we challenged mice with LPS to induce an inflammatory response. This intervention does not fully reproduce the low-grade inflammation observed in patients enrolled in CANTOS. Yet, low-dose LPS treatment is a well-established and reproducible method for experimental induction of inflammation that does not rely on altered metabolic conditions or genetic manipulations, unlike many other models (e.g., dyslipidaemia and obesity models, cytokine overexpressing animals), and thus avoids numerous variables in the experimental conditions. Furthermore, hsCRP—the inclusion criteria for the CANTOS trial—is not a reliable marker of inflammation in mice [53].

## 5. Conclusions

In conclusion, this study proposes reduced TF production by neutrophils undergoing NETosis as a novel mechanistic contributor to the reduction in CV events achieved by treatment with canakinumab in the CANTOS trial. The results affirm the tight interrelationship of inflammation with thrombosis, and point to processes and biomarkers that merit monitoring in further attempts to address residual cardiovascular risk with anti-inflammatory interventions.

## Figures and Tables

**Table 1:** Demographic and clinical characteristics of the study cohort.

	Whole Cohort (*n* = 66)	Low CRP * (*n* = 33)	High CRP (*n* = 33)	*p*
**Demographic**				
Age, years	66 ± 12	66 ± 12	65 ± 12	NS
Gender, m/f	46/20	23/10	23/10	NS
**Clinical and biochemical**				
Systolic BP ^†^, mmHg	130 ± 23	130 ± 26	123 ± 20	NS
Diastolic BP, mmHg	78±15	81±16	75±12	NS
BMI ^‡^, Kg/m^2^	25.5±3.6	25.2±3.6	25.8±3.6	NS
Hypertension	33 (50.0%)	17 (51.5%)	16 (48.5%)	NS
Diabetes	9 (13.6%)	5 (15.2%)	4 (12.1%)	NS
Active smokers	43 (65.2%)	10 (30.0%)	18 (54.5%)	NS
Total-c ^§^, mmol/L	4.93±1.12	5.15±1.26	4.70±0.93	NS
HDL-c ^∥^, mmol/L	1.22±0.31	1.24±0.27	1.21±0.34	NS
LDL-c ^#^, mmol/L	3.31±1.13	3.57±1.23	3.07±0.97	NS
Dyslipidaemia	33 (50.0%)	17 (51.5%)	16 (48.5%)	NS
**Medications**				
Aspirin	17 (25.8%)	9 (27.3%)	8 (24.2%)	NS
Clopidogrel	2 (3.0%)	1 (3.0%)	1 (3.0%)	NS
ACE-I ** or ARBs ^††^	17 (26.2%)	11 (33.3%)	6 (18.8%)	NS
β-blockers	10 (15.2%)	7 (21.2%)	3 (9.1%)	NS
Diuretics	6 (9.1%)	4 (12.1%)	2 (6.1%)	NS
Statins	12 (18.2%)	4 (12.1%)	8 (24.2%)	NS

* CRP: C reactive protein; ^†^ BP: blood pressure; ^‡^ BMI: body mass index; ^§^ Total-c: total cholesterol; ^∥^ HDL-c: high density lipoprotein cholesterol; ^#^ LDL-c: low density lipoprotein cholesterol; ** ACE-I: angiotensin converting enzyme inhibitors; ^††^ ARBs: angiotensin receptor blockers.

**Table 2:** Peripheral blood cell count.

	Vehicle	Anti IL *-1β	*p*
Total WBC ^‡^ (10^3^/mm^3^)	1.37 ± 0.13	1.38 ± 0.18	NS
Lymphocytes (%)	43.1 ± 5.2	32.3 ± 4.2	NS
Neutrophils (%)	51.4 ± 5.0	62.7 ± 4.2	NS
Monocytes (%)	5.5 ± 0.6	5.0 ± 0.7	NS
NLR ^†^	1.5 ± 0.3	2.7 ± 0.7	NS
Platelets (10^3^/mm^3^)	297.6 ± 44.2	312.8 ± 28.8	NS

* IL: interleukin; ^†^ NLR: neutrophil to lymphocyte ratio; ^‡^ WBC: white blood cells.

## Supplementary Materials

The following are available online at https://www.mdpi.com/2077-0383/8/12/2072/s1, Supplemental Figure S1. TF–DNA complex assay validation.
