# Supplementary material for: Interleukin-1β Mediates Arterial Thrombus Formation via NET-Associated Tissue Factor
Source: J Clin Med. 2019 Nov 26;8(12):2072. doi: 10.3390/jcm8122072 (PMC6947515; doi:10.3390/jcm8122072)
Supplement: Supplementary file 1 [file jcm-08-02072-s001.pdf]

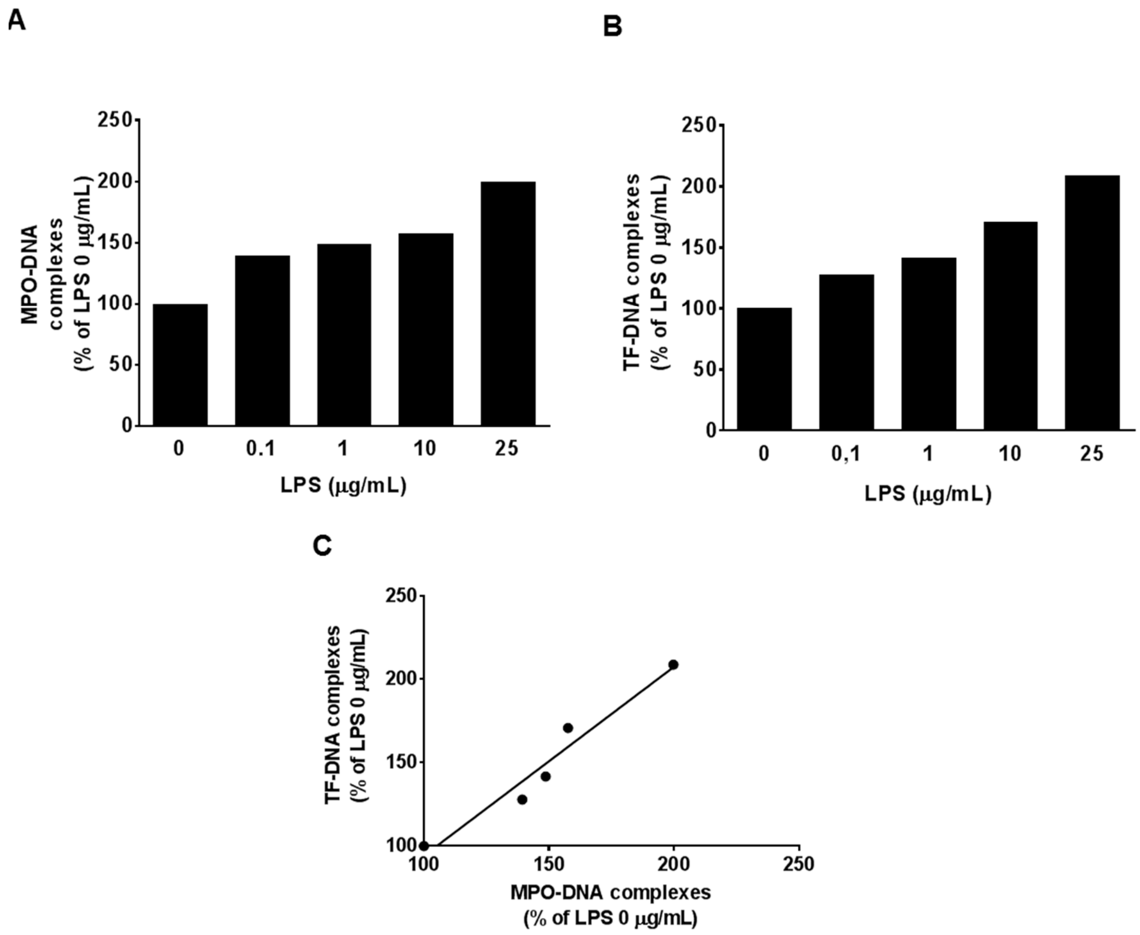

Supplemental Figure 1. TF-DNA complex assay validation. **(A)** As previously reported in the literature, MPO-DNA complexes in plasma dose-dependently increased after stimulation of the whole blood with increasing LPS dosages (0–25 μg/mL) (*n* = 1). **(B)** Levels of plasma TF-DNA complexes follow a similar dose-dependent trend being induced by higher LPS concentrations (*n* = 1). **(C)** Direct relationship between MPO-DNA and TF-DNA (*n* = 1). LPS = lipopolysaccharide, MPO = myeloperoxidase, TF = tissue factor.
